# The rate of early neurological deterioration occurring after thrombolytic therapy: A meta‐analysis

**DOI:** 10.1002/brb3.1210

**Published:** 2019-01-10

**Authors:** Xiaowen Hou, Wanli Chen, Haibin Xu, Zhi Zhu, Yuanyuan Xu, Huisheng Chen

**Affiliations:** ^1^ The Department of Neurology General Hospital of Shen‐Yang Military Region Shenyang China; ^2^ Group of Chronic Disease and Environmental Genomics China Medical University Shenyang China; ^3^ School of Materials Science and Engineering Shenyang Aerospace University Shenyang China

**Keywords:** early neurological deterioration, meta‐analysis, rate, stroke, thrombolytic therapy

## Abstract

**Objectives:**

The rate of early neurological deterioration (END) occurring after thrombolytic therapy is controversial. To explore a more precise estimation of the rate, a meta‐analysis was conducted in the present study.

**Methods:**

The relevant studies were identified by searching PubMed, EMBASE, and Cochrane Collaboration Database up to June 2018. The definition of END was prespecified according to the most commonly used definition: ≥4‐point increase in National Institutes of Health Stroke Scale between admission and 24 hr. The meta‐analysis was performed by using the STATA 12.

**Results:**

Eleven studies with a total of 3,539 subjects, including 373 patients with END and 3,166 patients without END, were collected. The pooled analysis showed that the rate of END occurring after thrombolytic therapy was about 11.0% (95% CI: 7.8%–14.3%). Subgroup analysis by continent showed that the rate of END occurring after thrombolytic therapy of patients in Asia (15.9%, 95% CI: 7.4%–24.5%) was higher than in Europe (7.6%, 95% CI: 4.9%–10.3%) and in North America (11.8%, 95% CI: 8.5%–15.0%). Subgroup analysis by onset to treatment time (OTT) displayed that the rate of END occurring after thrombolytic therapy was 5.4% (95% CI: 1.2%–9.5%), 15.6% (95% CI: 9.6%–21.5%), and 18.5% (95% CI: 11.2%–25.8%) for the patients whose OTT ≤120.0 min, from 120.1 to 179.9 min, from 180.0 to 270.0 min, respectively.

**Conclusion:**

The rate of END occurring after thrombolytic therapy is about 11.0%. This finding may provide a scientific reference for researchers to evaluate the efficacy and safety of thrombolytic therapy.

## 1 INTRODUCTION

Acute ischemic stroke (AIS) is the most common type of stroke and the vital cause of death and disability in the world (Murray et al., 2012; Sousa et al., 2017). Among the treatment methods of AIS, intravenous thrombolysis is recognized as the best option (Hacke et al., 2008; Powers et al., 2018). As the most commonly used thrombolytic drug, recombinant tissue plasminogen activator (rt‐PA) is considered to effectively improve the outcomes of AIS patients who are treated within 4.5 hr (Pan et al., 2014; Prabhakaran, Ruff, & Bernstein, 2015).

Although the outcomes of most AIS patients tend to be improved after intravenous thrombolytic therapy, there is still certain portion of patients experiencing early neurological deterioration (END). Unfortunately, no definition of END has been unified up to now. Most studies define END as ≥4‐point increase in National Institutes of Health Stroke Scale (NIHSS) between admission and 24 hr (Chi & Chan, 2017; Ong, Wong, Wu, & Su, 2017; Seners et al., 2017; Yang et al., 2016). Besides, some different definitions of END have been put forward by researchers. For instance, END has been defined as ≥1‐point increase in motor NIHSS or ≥2‐point increase in total NIHSS (Choi et al., 2016; Nam et al., 2017), ≥2‐point increase in NIHSS between admission and 24 hr (Kim et al., 2016), and ≥4‐point increase in NIHSS within 48–72 hr (Weimar et al., 2005). Because of the different definitions of END, the rate of END occurring after thrombolytic therapy reported in studies varied greatly.

To date, studies have been conducted extensively on the rate of END occurring after thrombolytic therapy. However, the overall rate cannot be easily estimated from individual publication with limited cases. Therefore, a meta‐analysis was conducted to evaluate the rate of END occurring after thrombolytic therapy.

## 2 METHODS

### 2.1 Searching strategy

A literature search from PubMed, EMBASE, and Cochrane Collaboration Database was conducted to identify the relevant studies up to June 2018. The following terms were used during the searching: (“early neurological deterioration” OR “END”) AND “thrombolysis” AND (“stroke” OR “cerebral infarction” OR “transient ischemic attack” OR “TIA”). No language or other restriction was used. Meanwhile, the references used in the eligible articles were also reviewed as sources to identify potential studies.

### 2.2 Inclusion and exclusion criteria

The studies included in the meta‐analysis had to meet the following criteria: (a) randomized trials or observational studies aiming at stroke or ischemic attack; (b) patients had received thrombolysis therapy; (c) sample size > 100; (d) providing the number of patients with END; and (e) having clear definition of END. In view of the fact that the definition of END is closely related to the rate of END, the definition of END was prespecified according to the most commonly used definition: ≥4‐point increase in NIHSS between admission and 24 hr.

Studies were excluded when they were as follows: (a) reviews, letters, case reports, protocols, or animal studies; (b) studies involving other definitions of END; and (c) studies providing insufficient information needed in the meta‐analysis.

### 2.3 Data extraction

Information was extracted independently by two experienced investigators, and any discrepancy was resolved by the third investigator. The authors whose studies did not provide adequate information should be contacted. The following data were collected as follows: first author's name, publication year, country, age, gender ratio, onset to treatment time (OTT), NIHSS at admission, thrombolytic drug type, thrombolytic drug dose, sample size, and the number of patients with END.

### 2.4 Quality assessment

Agency for Healthcare Research and Quality (Rockville, 2004) was used to assess the quality of the included studies. The assessment scale included 11 items. If an item was answered “Yes,” it would get one point; no point with answer “NO” or “UNCLEAR.” A study was considered high quality with 8–11 points; moderate quality with 4–7; and low quality with 0–3.

### 2.5 Statistical analysis

Data management and rate calculation of END were estimated by STATA 12 for Windows (Stata, College Station, TX, USA). Since meta‐analysis of rate generally has a significant heterogeneity, random effects model was chosen. A chi‐square test was used to examine heterogeneity between the included studies, and a *p* < 0.10 was considered statistically significant (Higgins, Thompson, Deeks, & Altman, 2003). To explore sources of heterogeneity, subgroup analyses were performed by continent (Asia, Europe or North America), OTT (≤120.0, 120.1–179.9 or 180.0–270.0 min), NIHSS at admission (≤4.0, 5.0–15.0, or 16.0–20.0), thrombolytic drug dose (0.6, 0.7, 0.8, 0.9 or 1.1 mg/kg), and study quality (high quality, moderate quality, or low quality). To evaluate the influence of each study on the pooled rate of END, sensitivity analysis was conducted. Publication bias was examined by using Begg's correlation and Egger's regression (Begg & Mazumdar, 1994; Egger, Davey, Schneider, & Minder, 1997). A *p < *0.05 was considered to be statistically significant.

## 3 RESULTS

### 3.1 Searching results

**Figure 1:**
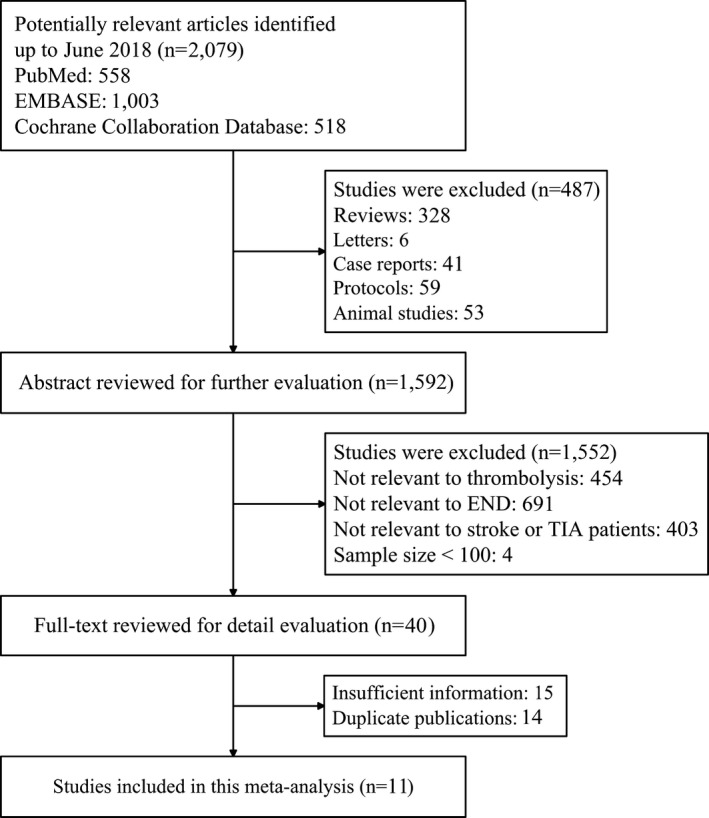
Flow diagram of study selection

According to our searching strategy, a total of 2,079 potential relevant studies were identified. Of these studies, 558 studies were from PubMed, 1,003 studies were from EMBASE, and 518 studies were from Cochrane Collaboration Database. Among these studies, 487 studies were excluded by reviewing titles. Then 1,552 studies were excluded by retrieving abstracts. Of the remains, 15 studies with insufficient information and 14 duplicate studies were removed. Finally, 11 studies met all the inclusion criteria. A flow diagram summarizing the whole process of selection procedures is shown in Figure 1.

### 3.2 Characteristics of the eligible studies

Eleven studies with a total of 3,539 subjects, including 373 patients with END and 3,166 patients without END, met the inclusion and exclusion criteria. Of these studies, there were 4 studies from Asia (Chi & Chan, 2017; Mori et al., 2012; Ong et al., 2017; Yang et al., 2016), 6 studies from Europe (Fiorelli et al., 1999; Hansen, Christensen, Havsteen, Ovesen, & Christensen, 2018; Seners et al., 2017, 2014; Simonsen et al., 2016; Zinkstok et al., 2014), and 1 study from North America (Saqqur et al., 2007). The baseline characteristics of all the included studies were shown in Table 1. Four studies provided the type and dose of the thrombolytic drug (Fiorelli et al., 1999; Mori et al., 2012; Ong et al., 2017; Yang et al., 2016), and the remaining studies provided insufficient information of the thrombolytic drug (Chi & Chan, 2017; Hansen et al., 2018; Saqqur et al., 2007; Seners et al., 2017, 2014; Simonsen et al., 2016; Zinkstok et al., 2014) (Table 2).

**Table 1:** Characteristics of the 11 studies included in the meta‐analysis

First author, published year	Country	Continent	Mean age/age range (yr)	Gender male %	Mean/median OTT (min)	Mean/median NIHSS at admission	END	Sample	*
Hansen et al (2018)	Denmark	Europe	70.0	55.7	NA	6.0^a^	27	361	8
Chi & Chan (2017)	China	Asia	70.0	52.4	158.6	13.3	93	323	6
Ong et al (2017)	China	Asia	66.2	57.7	116.2	14.1	21	274	6
Seners et al (2017)	France	Europe	69.4	46.7	155.0^a^	16.0^a^	22	120	8
Yang et al (2016)	China	Asia	59.0–72.3	55.5	191.6	3.4	20	108	8
Simonsen et al (2016)	Denmark	Europe	57.0–80.8	61.3	<180.0	7.0^a^	33	569	8
Zinkstok et al (2014)	Netherlands	Europe	66.7	50.0	115.0^a^	9.0^a^	11	320	5
Seners et al (2014)	France	Europe	69.1	53.0	156.0^a^	15.0^a^	33	309	7
Mori et al (2012)	Japan	Asia	72.0	62.7	144.6	12.8	56	566	9
Saqqur et al (2007)	Canada	North America	68.5	54.0	142.8	16.4	44	374	5
Fiorelli et al (1999)	Italy	Europe	NA	NA	NA	NA	13	215	8

END: early neurological deterioration; NA: not available; NIHSS: National Institutes of Health Stroke Scale; OTT: onset to treatment time.

a Median.

* Number of stars according to quality assessment scale.

**Table 2:** Presentation of thrombolytic drug of the 11 studies included in the meta‐analysis

First author, published year	Drug type	Drug dose (mg/kg)	END	Sample
Hansen et al (2018)	rt‐PA	NA	27	361
Chi & Chan (2017)	rt‐PA	NA	93	323
Ong et al (2017)	rt‐PA	0.6	3	71
		0.7	5	59
		0.8	6	88
		0.9	7	56
Seners et al (2017)	NA	NA	22	120
Yang et al (2016)	rt‐PA	0.6	9	46
		0.9	11	62
Simonsen et al (2016)	rt‐PA	NA	33	569
Zinkstok et al (2014)	NA	NA	11	320
Seners et al (2014)	rt‐PA	NA	33	309
Mori et al (2012)	rt‐PA	0.6	56	566
Saqqur et al (2007)	rt‐PA	NA	44	374
Fiorelli et al (1999)	rt‐PA	1.1	13	215

END: early neurological deterioration; NA: not available; rt‐PA: recombinant tissue plasminogen activator.

### 3.3 Quality assessment

The quality score of each study was presented in Table 1. There were 6 studies with high quality, 5 studies with moderate quality, and no study with low quality. The quality assessment showed reasonable study design and clear results. No study was excluded on grounds of quality.

### 3.4 Pooled analysis

**Figure 2:**
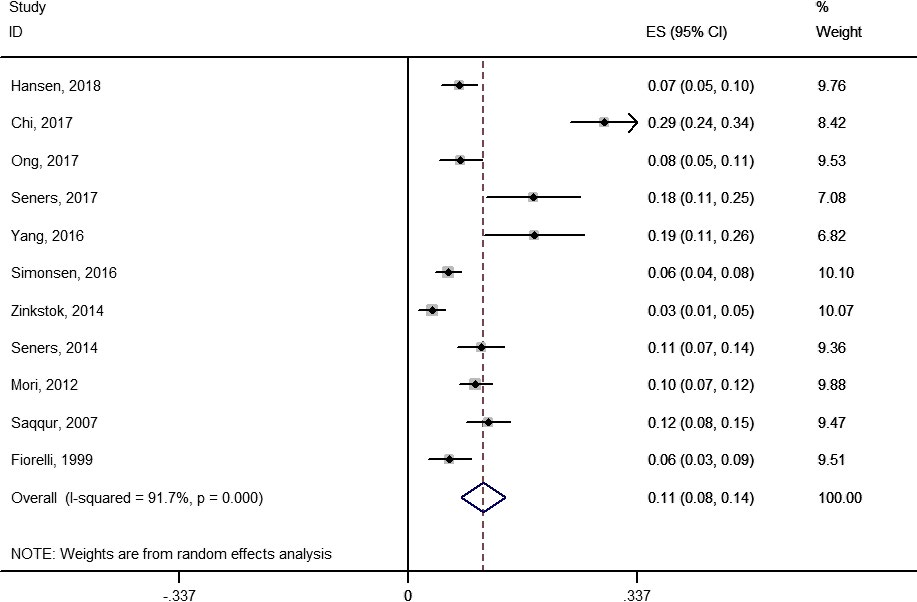
Forest plot of the rate of early neurological deterioration occurring after thrombolytic therapy

The rate of END occurring after thrombolytic therapy was calculated and ranged from 3.4% to 28.8% (Figure 2). The pooled rate of the 11 studies was about 11.0% (95% CI: 7.8%–14.3%). Substantial heterogeneity was found among the included studies (*p* < 0.001, *I* ^2^ = 91.7%).

### 3.5 Subgroup analysis

In the subgroup analysis, studies were categorized by continent, OTT, NIHSS at admission, thrombolytic drug dose, and study quality. The results of the subgroup analysis were showed in Table 3. In the subgroup analysis by continent, we found that the rate of END occurring after thrombolytic therapy in Europe was 7.6% (95% CI: 4.9%–10.3%), and in North America was 11.8% (95% CI: 8.5%–15.0%). However, the rate was higher in Asia (15.9%, 95% CI: 7.4%–24.5%). In the subgroup analysis by OTT, the rate of END occurring after thrombolytic therapy of the patients whose OTT ≤120.0 min, from 120.1 to 179.9 min and from 180.0 to 270.0 min was 5.4% (95% CI: 1.2%–9.5%), 15.6% (95% CI: 9.6%–21.5%), and 18.5% (95% CI: 11.2%–25.8%), respectively. In the subgroup analysis by NIHSS at admission, the results showed that the rate of END occurring after thrombolytic therapy of the patients whose NIHSS at admission was from 5.0 to 15.0 was 10.1% (95% CI: 6.1%–14.2%), and the rate of the patients whose NIHSS at admission was from 16.0 to 20.0 was 14.3% (95% CI: 8.0%–20.6%). However, the rate was higher in the patients whose NIHSS at admission ≤4.0 (18.5%, 95% CI: 11.2%–25.8%). Besides, the results of the subgroup analysis also showed that END rates were not associated with rt‐PA dose or study quality.

**Table 3:** The results of subgroup analysis

Subgroup	No. of study	Rate (%)	95% CI		Heterogeneity	
			Lower	Upper	*p*	*I^2 ^*(%)
Continent						
Asia	4	15.9	7.4	24.5	<0.001	94.8
Europe	6	7.6	4.9	10.3	<0.001	81.3
North America	1	11.8	8.5	15.0	NA	NA
OTT (min)						
≤120.0	2	5.4	1.2	9.5	0.026	79.7
120.1–179.9	5	15.6	9.6	21.5	<0.001	92.0
180.0–270.0	1	18.5	11.2	25.8	NA	NA
NIHSS at admission						
≤4.0	1	18.5	11.2	25.8	NA	NA
5.0–15.0	7	10.1	6.1	14.2	<0.001	93.8
16.0–20.0	2	14.3	8.0	20.6	0.093	64.6
Dose of rt‐PA (mg/kg)						
0.6	3	9.4	3.6	15.2	0.021	74.3
0.7	1	8.5	1.4	15.6	NA	NA
0.8	1	6.0	1.6	12.1	NA	NA
0.9	2	14.9	8.5	21.3	0.424	0.0
1.1	1	6.0	2.9	9.2	NA	NA
Study quality						
High quality	6	9.6	6.6	12.6	<0.001	80.2
Moderate quality	5	12.2	5.4	19.1	<0.001	95.8

NA: not available; NIHSS: National Institutes of Health Stroke Scale; OTT: onset to treatment time; rt‐PA: recombinant tissue plasminogen activator.

### 3.6 Sensitivity analysis

**Figure 3:**
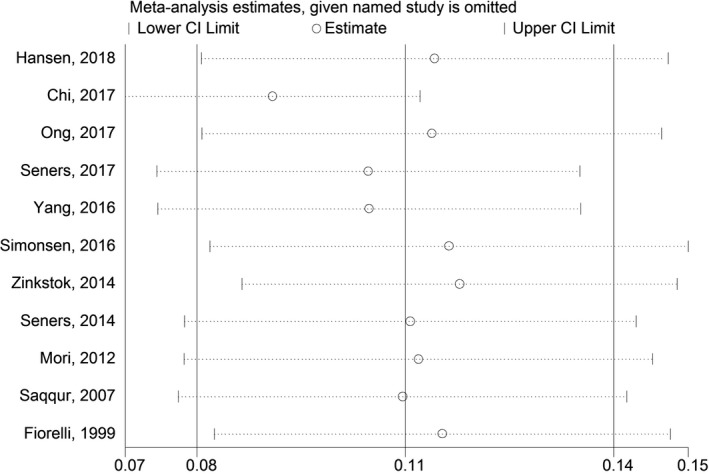
Sensitivity analysis of the rate of early neurological deterioration occurring after thrombolytic therapy

The sensitivity analysis was performed to evaluate the influence of each individual study on the pooled rate by omitting every single study. The analysis results reflected that the results were statistically stable and reliable (Figure 3).

### 3.7 Publication bias

Funnel plots showed slight asymmetry through visual inspection. However, Begg's test did not indicate significant publication bias in the meta‐analysis (*Z* = 1.25, *p* = 0.213).

## 4 DISCUSSION

To our knowledge, the paper has been the first meta‐analysis to evaluate the rate of END occurring after thrombolytic therapy. In the present study, 11 independent studies were included with a total of 3,539 subjects, comprised of 373 patients with END and 3,166 patients without END. The pooled analysis showed the rate of END occurring after thrombolytic therapy ranged from 3.4% to 28.8%, and the overall rate was about 11.0%. The results indicated that among one hundred patients who received thrombolytic therapy, eleven patients might experience END.

In the subgroup analysis by continent, the rate of END occurring after thrombolytic therapy was higher in Asia than in Europe and North America. One possible explanation for this result is the stroke pathophysiology in Asia differs from that in Europe and North America. In Asia, there are more arteriosclerotic large vessel stroke patients and large vessel occlusion patients who might have high NIHSS at baseline (Gorelick, Wong, Bae, & Pandey, 2008; Wong, 2006). In addition, the different programs for standard quality of care may also be responsible for this result. In the economically developed areas, the patients might receive better care quality than the patients in underdeveloped areas.

The “time is brain” concept has been introduced more than two decades which emphasizes the importance of time in treating acute stroke (Gomez, 1993). In our subgroup analysis by OTT, we found that the rate of END occurring after thrombolytic therapy was 5.4%, 15.6%, and 18.5% for the patients whose OTT ≤120.0 min, from 120.1 to 179.9 min, from 180.0 to 270.0 min, respectively. Therefore, OTT might significantly affect the rate of END occurring after thrombolytic therapy, and the rate might increase with the increase of OTT. Pulvers and Watson (2017) reported that emergency medical pathways, stroke symptomatology, patient and bystander behavior, patient health characteristics, and stroke treatment awareness might affect OTT, and reducing OTT might improve the stroke outcomes, which is consistent with our results.

National Institutes of Health Stroke Scale at admission is also a factor which has an important influence on the rate of END occurring after thrombolytic therapy. Some researchers suggested that NIHSS at admission in high level might increase the mortality and cause poor functional outcomes of patients receiving thrombolytic therapy (Kim et al., 2015; Lin, Chen, Hu, & Bai, 2018; Mazya et al., 2015). However, our results in this meta‐analysis did not conform to these conclusions. In the present subgroup analysis by NIHSS at admission, the rate of END occurring after thrombolytic therapy of the patients with NIHSS at admission ≤4.0 was highest among the three groups, which is inconsistent with most literatures. Two possible explanations for the phenomenon might be as follows. First, the study in which OTT ranged from 180.0 to 270.0 min was just the study in which NIHSS at admission was ≤4.0 (Yang et al., 2016), and OTT might have a greater impact on the rate of END than NIHSS at admission. Second, only one study was included in which NIHSS at admission was ≤4.0, so the results might be influenced by the inadequate sample size.

Some limitations of the meta‐analysis should be addressed. First, similar to other meta‐analyses of rate, high heterogeneity existed in the present study. Since there is no control group in the single arm studies, background difference in subjects cannot be well controlled and hardly be reduced by subgroup analysis, which often leads to great heterogeneity in the meta‐analysis of rate. Second, because the most commonly used definition of END (≥4‐point increase in NIHSS between admission and 24 hr) was adopted in this meta‐analysis, the conclusion in the study may be not suitable for other definitions of END. Third, sample size is still relatively small in this meta‐analysis, especially in the subgroup analysis. Therefore, studies with larger sample size are needed in the future. Finally, only publications were included in the present study, so we need more unpublished reports to expand our analysis. Despite the limitations, our meta‐analysis significantly increased the statistical power based on substantial data from different studies. The sensitivity analysis and publication bias outcomes both reflected that our results were statistically stable and reliable.

In summary, the meta‐analysis suggests that the rate of END occurring after thrombolytic therapy is about 11.0%. This finding may provide a scientific reference for researchers to evaluate the efficacy and safety of thrombolytic therapy. In order to reach a more definitive conclusion, a consistent definition of END is needed for future studies.

## CONFLICT OF INTEREST

The authors have no conflicts of interest to disclose.
